# Beauty isn't special: Comparing the information capacity of beauty and other sensory judgments

**DOI:** 10.1167/jov.23.7.6

**Published:** 2023-07-06

**Authors:** Maria Pombo, Denis G. Pelli

**Affiliations:** 1 Department of Psychology, New York University, New York, NY, USA; 2 Department of Psychology and Center for Neural Science, New York University, New York, NY, USA; 3 Center for Neural Science, New York University, New York, NY, USA

**Keywords:** mutual information, subjective beauty judgment, aesthetics, information theory, one-dimensional

## Abstract

Information theory (bits) allows comparing beauty judgment to perceptual judgment on the same absolute scale. In one of the most influential articles in psychology, Miller (1956) found that classifying a stimulus into one of eight or more categories of the attribute transmits roughly 2.6 bits of information. That corresponds to 7 ± 2 categories. This number is both remarkably small and highly conserved across attributes and sensory modalities. This appears to be a signature of one-dimensional perceptual judgment. We wondered whether beauty can break this limit. Beauty judgments matter and play a key role in many of our real-life decisions, large and small. Mutual information is how much information about one variable can be obtained from observing another. We measured the mutual information of 50 participants’ beauty ratings of everyday images. The mutual information saturated at 2.3 bits. We also replicated the results using different images. The 2.3 bits conveyed by beauty judgment are close to Miller's 2.6 bits of unidimensional perceptual judgment and far less than the 5 to 14 bits of a multidimensional perceptual judgment. By this measure, beauty judgment acts like a perceptual judgment, such as rating pitch, hue, or loudness.

## Introduction

When we judge the beauty of a perceived object, is beauty subject to the same rules as other perceptual judgments? Declaring the beauty of a sunset, or your favorite concert or meal, requires judging the perceptual experience. Is this any different from judging the color of the clouds, the pitch of a note, or the saltiness of food? More generally, does beauty belong in the study of perception? Importantly, perception is one of the original topics of neuroscience still remaining dominant. Does beauty require another chapter?

Based on what we know about beauty and perceptual judgments, it could go either way. Different senses have different organs in different body parts. One might imagine that judging beauty within a sense would behave like judging other attributes within that sense, especially given a similar task. On the other hand, unlike sensing color, pitch, or saltiness, beauty judgment is subjective, lacking a ground truth, highly variable across individuals and could even be considered an emotion. Emotional events are more memorable. Because categorizing requires memory of the categories, perhaps categories of beauty are particularly memorable and enable excellent categorization.

### Mutual information

To abstain from comparing apples to oranges, we compare beauty judgments to perceptual judgments on the same absolute scale. Information theory provides an appropriate framework for this comparison (Shannon, 1948). We can think of the human brain as an information channel, and we can measure how well a human rating is predictive of sensory information. The information transmitted through a channel is known as *mutual information*. Mutual information measures how well one can predict the input from the output, or vice versa, and can be measured in bits. One bit of information is the amount of information needed to decide between two equally likely alternatives. Two bits allow deciding among four alternatives, three between eight, and so on.

The mutual information carried by a perceptual judgment is highly informative of the dimensionality of its attribute. Miller (1956) found that humans have a low limit on information processing for unidimensional stimuli (stimuli varying on a single attribute), and a higher limit as stimulus dimensionality increases (stimuli varying on various attributes). Miller describes Pollack's experiments (Pollack, 1952; Pollack & Ficks, 1954) in which participants made absolute judgments of auditory tones. If the tones varied along a single dimension (e.g., pitch) participants could reliably categorize only six tones, corresponding to 2.6 bits. The limit is independent of the range of pitch used. If the tones vary along multiple dimensions (e.g., pitch, loudness, and duration), then participants can categorize 150 tones, corresponding to 7.2 bits. Moreover, the processing capacity in multidimensional categories expands way beyond 150. Adults can distinguish 10,000 images (Standing, 1973), and native-English-speaking college students can distinguish, on average, approximately 17,000 words (D'Anna, Zechmeister, & Hall, 1991) with few mistakes. Pollack's (1952) unidimensional tone categorization experiment is one of many examples Miller uses to exemplify his “magic” number, 7 ± 2 categories, or 2.6 ± 0.6 bits. This number is a human information processing limit and is conserved across sensory modalities (Beebe-Center, Rogers, & O'Connell, 1955; Eriksen & Hake, 1955a; Eriksen & Hake, 1955b). We use Miller's number as a benchmark to assess the dimensionality of a stimulus attribute.

If beauty judgments transmit 2.6 ± 0.6 bits of information, that would suggest that beauty judgments are one-dimensional. Significantly more bits would suggest that beauty is multidimensional. Some famous philosophical accounts suggest that beauty is special, such as Kant's idea that pleasure in beauty is unlike sensuous gratification or Sibley's claim that detecting aesthetic properties requires a specialized sensitivity (Kant, 1790; Sibley, 1959; cf., Zangwill, 2022). Other philosophers talk about beauty as “unity-in diversity,” which sounds like projecting the many stimulus dimensions into one perceived dimension. As described by Diessner and colleagues (2018), “unity-in-diversity means that a variety of elements are organized into a meaningful whole” (p. 378). Plato describes beauty as parts that “fit harmoniously into a seamless whole” (cf., Etcoff, 2000, p. 15), and Hutcheson as “uniformity in variety” (cf., Dickie & Sclafani, 1977, p. 14). Wittgenstein (1938) also alluded to this idea in his description of “harmony” in beauty judgments. Scientifically, one can treat beauty judgments as perceptual judgments (Pombo & Pelli, 2022a). In a multidimensional scaling of beauty comparisons, Wu, Brielmann, & Pelli (2019) found that beauty is one dimensional. So, is beauty incomparable, or is it like other types of perceptual judgment?

### Beauty and information theory

The aesthetics literature occasionally cites information theory. Moles (1957) considered engagement with art as communication. Although today perception is often thought of as information transmission from stimulus to receiver, in art, Moles considers the information transmission from artist to observer. And the message, or art piece, is an orderly pattern of elements selected from a repertoire of possible elements. A song is composed of orderly elements of musical notes or sounds, and a poem is an orderly composition of letters and words (Burt, 1966). Moles goes on to claim that the amount of information the observer of an artwork receives closely relates to the intensity of what they feel (Biederman & Vessel, 2006; Rashevsky, 1967). Like Moles, Berlyne (1971, 1974) presents a qualitative account of information transmission while viewing art. He claims that works of art have four information sources: semantic, expressive, cultural, and syntactic. He notes that these elements may give a stimulus hedonic value through their “collative” properties: novelty, surprisingness, complexity, ambiguity, and puzzlingness. Specifically, he notes that the relationship between collative properties and preference follows a Wundt curve, or an upside-down U-shape.

Ever since Berlyne's theory on the relationship between preference and complexity, many have used Shannon entropy (Shannon, 1948), a hallmark of information theory, to operationalize complexity. Whether this relationship between complexity and preference holds is still a matter of debate among aesthetics researchers in the auditory (see Chmiel & Schubert, 2017, for a review) and visual (see Nadal, Munar, Marty, & Cela-Conde, 2010, for a review) domains. Beyond using information theory as an operationalization tool, some propose that pleasure stems from an inherent desire to acquire information (Biederman & Vessel, 2006; Grzywacz & Aleem, 2022; Ripollés et al., 2016). Others even propose to limit the amount of information in artworks because humans can only process a given number of bits at a time (Franke, 1977). However, these accounts refer to beauty *experiences* as opposed to beauty *ratings*, and how much information beauty ratings convey remains unknown.

### Current study

The current study assesses the mutual information of beauty judgment. Using a simple image categorization paradigm, we measure mutual information (i.e., how well each beauty rating of an image predicts that participant's average beauty rating of that image). We then compare the observed mutual information to Miller's benchmark of 2.6 bits as an indication of the dimensionality of beauty rating. Overall, and for the first time, we are able to compare beauty ratings to perceptual ratings along the same absolute scale, assessing whether beauty rating is special or not.

## Methods

### Participants

We recruited 50 participants through Prolific Academic (https://prolific.co/) to take part in our experiment. Twenty-five identified themselves as female, 24 as male, and one as “other.” Their ages ranged from 18 to 70 (*M* = 29, SD = 10). All participants were U.S. nationals, spoke English as their first language, and indicated having normal or corrected-to-normal vision. All participants gave informed consent in accordance with the Declaration of Helsinki. This experiment was approved by the New York University Committee on Activities Involving Human Subjects (IRB-FY2019-2456).

### Procedure

After providing informed consent and completing demographic questions about age and gender, participants completed four experimental blocks in random order. Each block contained five repetitions of three, four, six, or 10 different images. Thus there was a total of 15, 20, 30, or 50 stimuli in each block. Memory of the stimulus rating across image repetitions is not a concern because recall memory does not significantly affect the variance of beauty ratings (Pombo, Brielmann, & Pelli, 2023). In each trial, participants were asked to rate, on a scale from 1 (not at all) to 10 (very much), how much beauty they felt from looking at an image. The order of the images in each block was pseudorandomized, with the constraint of never showing the same image twice in a row. All images were presented at the center of the screen on a white background. The scale was composed of black radio buttons that became gray upon a click and had the corresponding numerical rating on the bottom. After selecting their rating, participants had to press a “continue” button in the bottom right corner to see the next image.

In this paradigm, Pollack and the other studies reviewed by Miller manipulate source entropy by varying the number of unique stimuli. The general result is that as source entropy is increased, mutual information is equal to source entropy until it reaches a plateau. We are following that paradigm and vary image number over a sufficient range to estimate where the plateau begins.

### Stimuli

**Figure 1.**
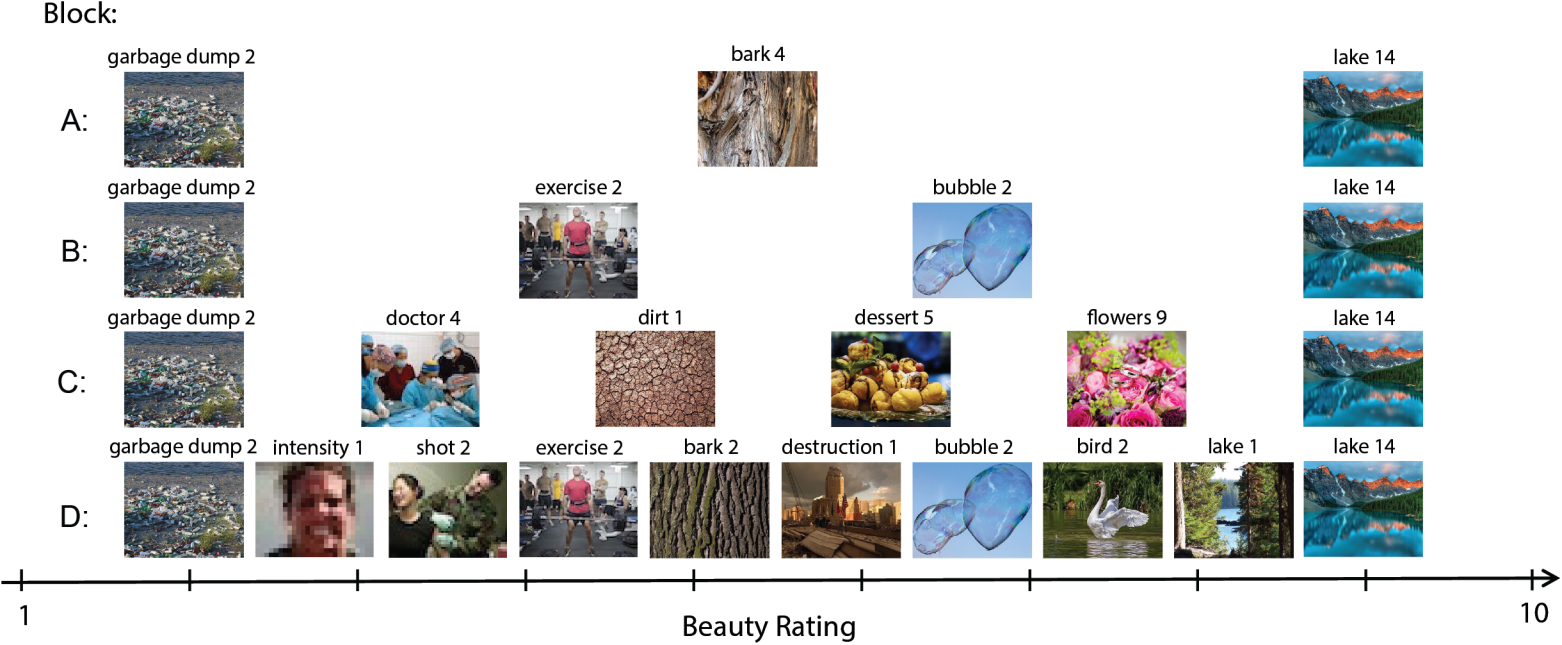
Distribution of average beauty ratings in each block. The images were selected based on their crowd-sourced beauty rating (Brielmann & Pelli, 2019). The mean beauty rating for each image across participants in our study closely matches the crowd-sourced ones. Each participant saw each image five times in each block. The order of images and blocks was randomized. Labels are consistent with the image dataset.

We selected 15 images from the OASIS dataset (Kurdi, Lozano, & Banaji, 2017). Each OASIS image shows a person, scene, animal, or thing. We selected the images based on the beauty ratings collected by Brielmann and Pelli (2019) after converting their ratings from a seven-point scale to a 10-point scale. A 10-point scale allowed us to have enough categories so that the scale itself would not limit our measure of mutual information. Previous beauty ratings were converted into this scale to allow comparison. We selected the image with the highest mean beauty rating (rated 9.8 out of 10) and the image with the lowest beauty ratings that did not contain any violent or graphic content (rated 2.7 out of 10). All blocks included both of those images, and the remaining images in each block were uniformly distributed between 2.7 and 9.8. Following Pollack's experiments with pure tones (1952), all blocks included the images with extreme ratings so the range of beauty ratings remained consistent across blocks. All participants saw the same images. Figure 1 shows the stimuli we used in each block and their distribution along the beauty scale. The image display size was 500 × 400 px, which, on a 15-inch 2880 × 1800 px display, corresponds to about 5.3° × 6.3° of visual angle for an observer at a 50 cm distance from the screen.

### Apparatus

The experiment was programmed on PsychoPy version 2021.2.3 (Peirce et al., 2019) and delivered online by the Pavlovia server (https://pavlovia.org). All participants used a computer to complete the experiment (37 used Win32, 12 used MacIntel, and one used Linux x86_64).

### Analysis

All analyses were conducted using R (version 4.0.5) in RStudio. For each participant and image, we calculated the mean beauty rating (of the five ratings provided per image per block), which we rounded to the nearest integer. We considered this to be the input. We then calculated the mutual information between this input and each individual beauty rating (output).

Mathematically, the mutual information *I*(*X* : *Y*) between an input *X* and an output *Y* is defined as follows:

(1) $$\begin{array}{ccc} & & \;I\left(X:\;Y\right)=\sum_{xɛX}\sum_{xɛY}p\left(x,\;y\right)\log_{2}\left(\frac{p\left(x,\;y\right)}{p\left(x\right)\;p\left(y\right)}\right)\; \end{array}$$

*p*(*x*, *y*) corresponds to the joint probability of x and y and *p*(*x*) and *p*(*y*) correspond to the marginal probabilities. These probabilities can be calculated using a contingency table.

To calculate the mutual information, we used the *infotheo* package in R (Meyer, 2014). The data and code experiments can be found here: https://osf.io/f2s8v/. We verified our calculation by comparing the estimated mutual information with that from a similar package in Python (Pedregosa et al., 2011).

## Results

**Figure 2.**
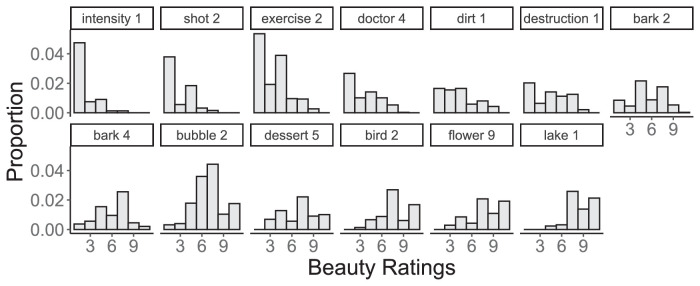
Distribution of beauty ratings for each image. Labels are consistent with Figure 1 and OASIS.

For each participant and image, we calculated the mean beauty rating as the input. We then calculated the mutual information between this input and each individual beauty rating (output) as described above. The input and output distributions were very similar, both with a mean of *M* = 5.3, and standard deviations of 3.34 and 3.35 respectively. Both the input and output span the entire range of beauty values (1 to 10). The distribution of beauty ratings for each image is shown in Figure 2.

**Figure 3.**
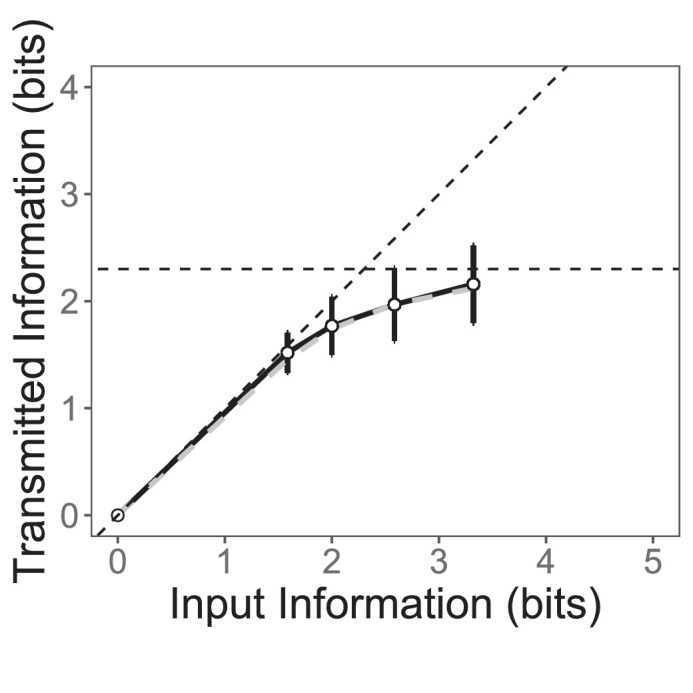
Mutual information as a function of the information of the source. The input information refers to log_2_ (number of images). The error bars correspond to the standard deviation. The origin is included because no information should be transmitted if the source has no information. Gray line displays replication results. See Supplementary Tables S1.1 and S2.1 for exact point values.

Like Miller, we found that increasing the input information (by increasing the number of distinct stimuli), increased the mutual information, approaching an asymptote of 2.3 bits. This asymptote is slightly higher than the rightmost point on the graph (2.2 ± 0.4 bits) (Figure 3). The 2.3 bits corresponds to somewhere between 4 and 7 categories. This value is within Miller's estimate of 2.6 ± 0.6. The 4 to 6 categories conveyed by beauty judgment are close to the 7 ± 2 categories of unidimensional and far less than the 32 to 8,000,000 of a multidimensional perceptual judgment.

In addition, we wanted to ensure that the crowd-sourced beauty ratings we used to select the stimuli did not differ from the mean beauty ratings across participants in our sample. The strong correlation between ratings, *r =* 0.988, *t*(21) = 29.82, *p* < 0.001, 95% CI = 0.972-0.995, validates our stimuli choices.

The data points we used in our calculation of mutual information are not independent. The mean beauty rating contains the individual beauty rating, so we took a leave-one-out approach (for an example of leave-one-out in aesthetics, see Vessel, Maurer, Denker, & Starr, 2018) to ensure that this dependence did not inflate our results. We repeated the analysis using mean beauty ratings that excluded that individual beauty rating (i.e., the mean is now calculated from the other four beauty ratings of that stimulus in that block). The results are very similar, and as source entropy increases, mutual information also reaches a plateau at 2.2 ± 0.4 bits (see Supplementary Table S1.2).

### Replication

To ensure that our results were not exclusive to the 15 chosen images, we directly replicated the experiment with 50 new participants. We selected a different set of OASIS images and collected beauty ratings. Our results directly replicate our original experiment and indicate that as source entropy increases, mutual information increases and plateaus at 2.1 ± 0.3 bits (See Supplementary Materials).

## Discussion

This study is the first to compare subjective and perceptual judgments on the same absolute scale. We adapted Pollack's experiment (1952) on pitch judgments to calculate the mutual information of beauty judgments of everyday images. By using each individual's mean rating for each image as the participant's “ground truth,” and using it to predict each rating, we estimated that beauty judgments transmit 2.3 bits of information. Thus beauty judgments behave a lot more like perceptual unidimensional judgments than multidimensional judgments (which transmit 3 to 14 bits).

Our results offer insight into the relationship between beauty and perception. At least in how much information they carry, beauty judgments are like unidimensional perceptual judgments. According to our results, it is conceivable that beauty judgments are just like any other perceptual judgments, except with high individual differences (Pombo & Pelli, 2022a). At the very least, beauty judgments are more than merely subjective or spiritual inklings. They belong within perception research.

In his article, Miller (1956) describes both the information capacity limits examined herein, as well the span of short-term memory, which he deems to also be 7 ± 2 items or “chunks.” However, he calls the resemblance between the absolute judgment limit and the memory span limit a coincidence. Whether the memory limit span is in fact 7 ± 2 is greatly disputed (see Baddeley, 1994; Cowan, 2001), and researchers question whether similar underlying mechanisms can explain both accounts (Cowan, 2015). Nevertheless, Miller's categorization experiments use a classic identification paradigm to study sensory limits. When perception scientists study sensory limits, they typically use only a few similar stimuli. When memory researchers study memory limits, they typically use many diverse stimuli (Carney, Widin, & Viemeister, 1977). The absolute judgment experiments reviewed by Miller span the range from few to many stimuli. These experiments avoid discrimination limits by choosing categories that are more than one JND (just-noticeable difference) apart. Once you get away from the JND limit, the mutual information is independent of stimulus spacing and similar across all senses. Hence, Miller offers a 7 ± 2 category limit as a parsimonious account of all the results.

That beauty judgments transmit 2.3 bits of information, near Miller's (1956) one-dimension benchmark, suggests that beauty judgment too is one-dimensional. Unidimensionality of beauty would have important implications for beauty-based decision-making. Our everyday decisions involve a variety of factors: what shirt we pick out from our closet every morning depends on the weather, how the shirt fits us, and the type of impression we want to convey that day, among other things. The meal we pick at a restaurant depends on what the menu says, how hungry we are, our dietary restrictions, others’ recommendations, and perhaps our internal emotional state. To make decisions, we must be able to represent our choices in a common unit of measurement (Padoa-Schioppa, 2011), a process referred to as “valuation” (Rangel, Camerer, & Montague, 2008). If beauty judgments are one dimensional, then beauty judgments could be that common scale. Beauty judgments may allow us to combine variability along many dimensions to rank, sort, and compare alternatives.

Suggesting that beauty judgment is one dimensional does not trivialize the feeling of beauty. Beauty *experiences* are special. In fact, our most beautiful memories are often of experiences that define us. However, recognizing that beauty *judgments* are not special and resemble other perceptual judgments facilitates the empirical study of beauty. In the same way that we can calculate how much information beauty judgments transmit, we can calculate their variance within and across individuals (Brielmann & Pelli, 2019; Chen et al., 2022; Leder, Goller, Rigotti, & Forster, 2016; Vessel et al., 2018) and model them (Aleem, Correa-Herran, & Grzywacz, 2020; Brielmann & Dayan, 2022).

## Conclusion

In this study we compare beauty judgment to perceptual judgments on an absolute scale. By measuring the mutual information of beauty judgments, a metric originating from information theory, we estimate the dimensionality of beauty judgment. Miller (1956) discovered that the transmission of about 2.6 bits is a remarkably well-conserved aspect of one-dimensional judgment, whereas multidimensional judgments transmit more bits (3 to 14). We asked participants to categorize the beauty of an image and varied the number of categories. We found that the amount of information transmitted by each beauty rating increases with the number of categories, with an asymptote of 2.3 bits. This is similar to the 2.6 ± 0.6 for one-dimensional perception judgment and much smaller than the 3 to 14 bits for multidimensional perception judgment, which together suggest that beauty judgment is one-dimensional. In our results, beauty judgment is like perceptual judgment. Overall, we position beauty judgments in the context of perception research and identify how beauty's unidimensionality makes beauty judgments a suitable basis for decision-making.

## Supplementary Material

Supplement 1
