# Supplementary material for: Beauty isn't special: Comparing the information capacity of beauty and other sensory judgments
Source: J Vis. 2023 Jul 6;23(7):6. doi: 10.1167/jov.23.7.6 (PMC10337797; doi:10.1167/jov.23.7.6)
Supplement: Supplement 1 [file jovi-23-7-6_s001.pdf]

Supplementary Materials

S1. Tables

Table S1.1 Summary statistics of the mutual information of beauty judgment

Number of Images	Information of Source (bits)	Mean mutual information (bits)	Standard deviation of mutual information (bits)
3	1.58	1.52	0.19
4	2	1.77	0.27
6	2.58	1.97	0.34
10	3.32	2.16	0.36

Table S1.2. Summary statistics of the mutual information of beauty judgment - Leave-one-out analysis

Number of Images	Information of Source (bits)	Mean mutual information (bits)	Standard deviation of mutual information (bits)
3	1.58	1.54	0.19
4	2	1.79	0.26
6	2.58	2	0.32
10	3.32	2.17	0.36

S2. Replication

Methods

50 new Prolific participants took part in our replication experiment. 26 identified themselves as female, 22 as male, and two as “other”. Their ages ranged from 18 to 79 (*M* = 36.5, *SD* = 14). We selected a non-overlapping set of 15 OASIS images following the same criteria as our original experiment. The experimental design and analysis remained identical. The images, their beauty ratings, and the analysis is available here: https://osf.io/f2s8v/?view_only=b415abe1ff144c1b8553ea28719e1d8e.

Results

Once again, we found that increasing the input information (by increasing the number of distinct stimuli) increased the mutual information, approaching an asymptote of 2.3 bits (Fig. S1). For 10 stimuli, mutual information is 2.1 ± 0.3, again within Miller’s estimate of 2.6 ± 0.6. Results are very similar to those presented in Figure 3 of the main manuscript.

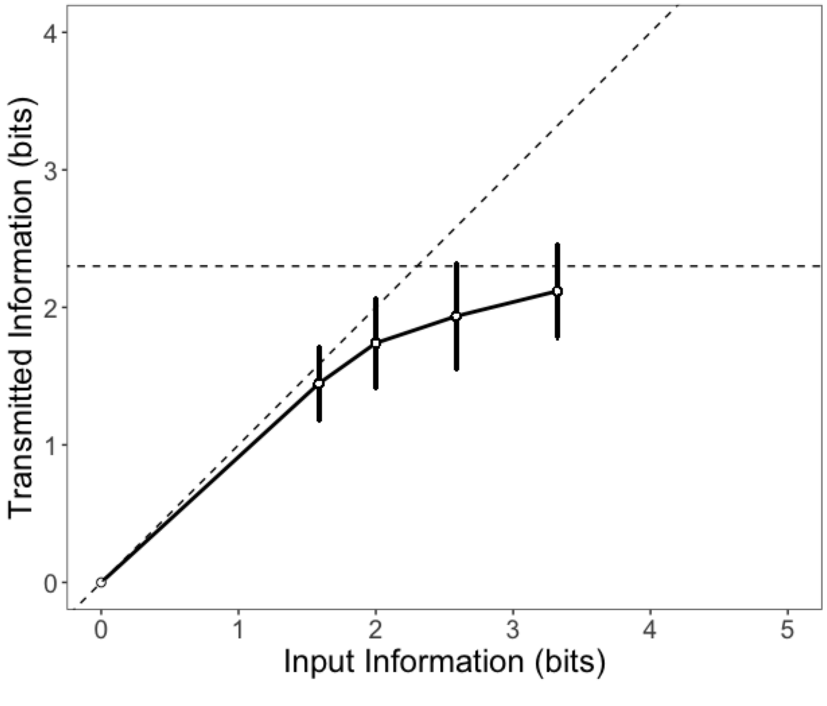

Figure S1. Mutual information as a function of the information of the source. The input information refers to $\log_2$(number of images). The error bars correspond to the standard deviation. The origin is included since no information should be transmitted if the source has no information.

Table S2.1. Summary statistics of the mutual information of beauty judgment - Replication

Number of Images	Information of Source (bits)	Mean mutual information (bits)	Standard deviation of mutual information (bits)
3	1.58	1.45	0.27
4	2	1.74	0.33
6	2.58	1.97	0.39
10	3.32	2.12	0.34
